# Joint Adaptive Sampling Interval and Power Allocation for Maneuvering Target Tracking in a Multiple Opportunistic Array Radar System

**DOI:** 10.3390/s20040981

**Published:** 2020-02-12

**Authors:** Qinghua Han, Minghai Pan, Weijun Long, Zhiheng Liang, Chenggang Shan

**Affiliations:** 1 College of Information Science and Engineering, Zaozhuang University, Zaozhuang 277160, China; uzz_scg@163.com; 2 Ministry of Education Key Laboratory of Radar Imaging and Microwave Photonics, Nanjing University of Aeronautics and Astronautics, Nanjing 211106, China; panmh@nuaa.edu.cn; 3 The 14th Research Institute of China Electronics Technology Group Corporation, Nanjing 210039, China; chinacohit@163.com; 4 Department of Precision Instrument, School of Mechanical Engineering, Tsinghua University, Beijing 100084, China; liangzhiheng@mail.tsinghua.edu.cn

**Keywords:** joint adaptive sampling interval and power allocation (JASIPA), chance-constraint programming (CCP), maneuvering target tracking (MTT), best-fitting Gaussian (BFG), Cramér-Rao lower bound like (CRLB-like)

## Abstract

In this paper, a joint adaptive sampling interval and power allocation (JASIPA) scheme based on chance-constraint programming (CCP) is proposed for maneuvering target tracking (MTT) in a multiple opportunistic array radar (OAR) system. In order to conveniently predict the maneuvering target state of the next sampling instant, the best-fitting Gaussian (BFG) approximation is introduced and used to replace the multimodal prior target probability density function (PDF) at each time step. Since the mean and covariance of the BFG approximation can be computed by a recursive formula, we can utilize an existing Riccati-like recursion to accomplish effective resource allocation. The prior Cramér-Rao lower boundary (prior CRLB-like) is compared with the upper boundary of the desired tracking error range to determine the adaptive sampling interval, and the Bayesian CRLB-like (BCRLB-like) gives a criterion used for measuring power allocation. In addition, considering the randomness of target radar cross section (RCS), we adopt the CCP to package the deterministic resource management model, which minimizes the total transmitted power by effective resource allocation. Lastly, the stochastic simulation is embedded into a genetic algorithm (GA) to produce a hybrid intelligent optimization algorithm (HIOA) to solve the CCP optimization problem. Simulation results show that the global performance of the radar system can be improved effectively by the resource allocation scheme.

## 1. Introduction

### 1.1. Background and Motivation

The opportunistic array radar (OAR), proposed by the United States Naval Postgraduate School (NPS), is a new system radar for the next generation naval stealth destroyer DD(X) [1,2,3]. In the OAR system, the stealth of the platform is taken as the core and the digital array is regarded as the base, and then the array elements and the transmit/receive (T/R) modules are placed arbitrarily and aperiodically at available open areas over the entire 3-D space of carrier platforms [4,5]. Due to the unique structure of the antenna array, the resource management of OAR is flexible. On the other hand, the maneuvering target tracking (MTT) plays a vital part for various commercial and military applications and receives plenty of attention [6,7,8,9]. For example, the application areas include battlefield surveillance, air traffic control, air defense, and fire control. Hence, the resource management for MTT in the OAR system is a significant and worthwhile research direction.

So far, many studies has been done to enhance the utilization efficiency of a scarce radar resource in the target tracking process [10,11,12,13,14,15,16]. Reference [10] proposes a sensor selection policy for static target location in distributed multiple-radar architectures. This policy is formulated in a combinatorial optimization framework as a knapsack problem (KP) to obtain a performance level with the lowest cost. Referring to the Bayesian Cramér-Rao lower bound (BCRLB) as a criteria, Reference [11] puts forward a joint power and beams allocation algorithm for multiple target tracking in a co-located multiple input multiple output (MIMO) radar system. Besides, by incorporating an information reduction factor (IRF), the BCRLB is efficiently computed with a measurement origin uncertainty (MOU) and the radar resource is allocated to the targets reasonably in clutter [12]. A joint node selection and power allocation strategy is developed with the objective of tracking multiple targets in Reference [15]. In order to improve the worst-case tracking accuracy with multiple targets, the proposed mechanism implements the optimal resource allocation based on the feedback information in the tracking recursion cycle. In Reference [16], the resource allocation problem concerns the sensor subset, power, and bandwidth. The three-variable optimization problem is simplified by deriving the relationship between the optimal power allocation vector and the bandwidth allocation vector. This algorithm can help achieve better performance within the same resource constraints.

The previously mentioned work provides us with an opportunity, and helps to deal with the resource management problem. However, this work only considers the resource management problems of a uniform moving target [11,12,13,14,15,16]. In the actual battlefield environment, such a favorable circumstance is always an unrealistic assumption. In military applications, the target aircrafts typically fly intelligently in order to achieve their objectives. Even they have electronic equipment that can measure the active sensor’s attempts to track them and may respond accordingly. Therefore, an intelligent resource allocation framework is presented herein for tracking a maneuvering target with Markovian switching dynamics (MSD means that the current target state only depends on the previous target states) in multiple OAR systems. This is a hybrid estimation problem where it is required to estimate sequentially both the continuous-valued target state and the discrete-valued switching model variable. There are several existing error bounds that deal with the hybrid system estimation problem. Examples are Bhattacharyya, Bobrovsky-Zakai, and Weiss-Weinstein lower bounds [17]. However, the derivations of these bounds are extremely complex and their implementation is very involved [18,19]. In addition, the direct application of the BCRLB recursive formula for nonlinear filtering [20] would lead to differentiation of terms involving the discrete-valued model variable. Hence, an approximation to the BCRLB for MSD is considered in this case [21,22,23]. The essence of this technique is to replace the multimodal prior target probability density function (PDF) with a best-fitting Gaussian (BFG) distribution at each time step. The mean and covariance of BFG distribution can be computed through a recursive formula.

In a traditional multiple radar system, prior knowledge is not utilized for a resource allocation scheme. In general, the sampling interval is fixed and the transmitted power of spatially separate radars is equal. Such an operation will definitely result in the inadequate use of the limited time and power resources. In terms of time resource, if the sampling interval is too long, it may lead to a large tracking error, or even lose the target. If the sampling interval is too short, the frequent beam illumination is a waste of the radar resource. Through the BFG approximation, we could determine when to transmit beams to acquire data of the tracked targets. The prior Cramér-Rao lower boundary (prior CRLB-like) can be set as a criterion for an adaptive sampling interval [11]. As a consequence, more tracking tasks can be maintained and more radar resources can be applied to track initiation [24]. Regarding the power resource, based on the prior CRLB-like, the BCRLB-like can be calculated as an error measurement contributing to the intelligent power allocation [20].

Generally speaking, in the traditional work of radar resource management, the radar cross section (RCS) of the illuminated target is considered to be a determined value at each sampling instant [11,12,13,15,16]. Due to the effects of the identity, attitude, position, aspect angle, wave length, and polarization, the RCS of the next sampling instant is uncertainty [25], i.e., the RCS to be estimated obeys a certain distribution. To adequately disclose the uncertainty of RCS, we regard it as a random variable. On account of the randomness of constraint conditions, we introduce chance-constraint programming (CCP) to handle the uncertainty by guaranteeing that the stochastic constraint holds at a specific confidence level [26]. When the confidence level is equal to one, we need to provide a radar resource than in other cases to satisfy the constraints. Yet, it can achieve better tracking performance. However, it is not cost-effective to spend superfluous radar resource on the extremely low probability incident that the RCS takes values at the lower boundary of the distribution range. In addition, to obtain an acceptable tracking error, the confidence level cannot be low either. The confidence level is decided by the environments and desired tracking performance. Therefore, the tracking accuracy could be adjusted flexibly conditioned on different confidence levels. Since we abandon the extreme case that satisfies the constraints conditioned on a very low probability, the resource consumption is reduced strikingly to maintain more tracks.

Through the previously mentioned analysis, we propose a joint adaptive sampling interval and power allocation scheme based on CCP for MTT using BFG in multiple OAR systems. The BFG distribution is presented to replace the multimodal prior target PDF at each time step. In order to realize an adaptive sampling interval, the prior CRLB-like is calculated to decide when to illuminate the target. In conjunction with the data Fisher information matrix (FIM) obtained from distributed radars, the approximation of BCRLB is derived for a maneuvering target with MSD, and it is named BCRLB-like. Conditioned on a specified confidence level, we adopt the CCP to package the deterministic resource management model as the final uncertain model. The whole algorithm can be seen as an intelligent response of a radar system to MTT by perceiving environments. The BCRLB-like is to measure the error for target state estimation, and it provides us a criterion to pre-allocate the radar resource. The optimization problem can be solved by a hybrid intelligent optimization algorithm (HIOA) integrating a stochastic simulation and genetic algorithm (GA).

### 1.2. Main Contributions and Innovations

The main contributions and innovations of this paper are as follows.

(1) The BFG approximation is employed so as to allocate the radar resource conveniently. Due to the diversities of motion states of maneuvering targets, it is difficult to predict the target state, and the radar resource allocation has no referenced criterion at the next sampling instant. Guaranteeing that the state vectors have the same mean and covariance under different models, the BFG approximation is introduced to replace the multimodal prior target PDF at each time step. The target state can be predicted easily by a single motion equation, and then accomplish the resource pre-allocation.
(2) A joint adaptive sampling interval and power allocation scheme is presented. The prior CRLB-like as a measurement criteria is compared with the upper boundary of the given tracking error threshold to determine the next optimal sampling interval. The tracking BCRLB-like is computed for power allocation among the distributed radars. The diagonal elements of BCRLB-like provide a referenced boundary on the variances of the estimation of the target’s hybrid state.
(3) The CCP is brought in to handle the uncertainty of target information in a resource management model. The target RCS is regarded as a random variable. The CCP balances the radar resource and the tracking performance by adjusting the confidence level. If the target environment is simple or the tracking performance requirement is low, the confidence level could be lowered appropriately to save more resources for other tasks.

The remainder of this paper is structured as follows. In Section 2, we provide the system model for a linear jump Markov system. In Section 3, the CCP model is formulated. By introducing the BFG approximation, the BCRLB-like is derived as the inverse of the Bayesian information matrix (BIM) for MTT. This model integrates the adaptive sampling interval and power allocation in multiple OAR systems. Section 4 proposes the HIOA to solve the model, and two algorithms of target state estimation are given in this section. The simulation results and corresponding analysis are provided in Section 5 to verify the effectiveness of the proposed resource allocation scheme. Lastly, we conclude this paper in Section 6.

## 2. System Model

**Figure 1:**
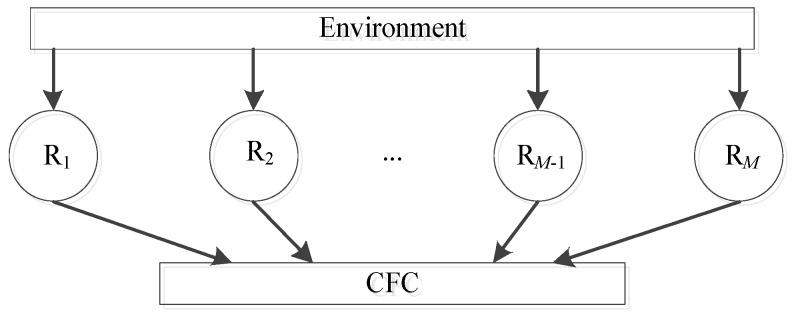
Centralized fusion architecture.

Suppose a two-dimensional multiple radar system including *M* (*M* ≥ 2) distributed monostatic radars. The *q*th radar is located at (*x_q_*, *y_q_*) (*q* = 1, 2, …, *M*). We assume that: (1) the carrier frequency of each radar is different. (2) There is only a matched filter for the respective transmitted signal. In terms of the two assumptions, each radar receives all the echo signals from the target, but the exclusive matched filter can filter out the echo signals transmitted by the other radars. Hence, each radar operates in a monostatic way. In addition, the centralized tracking is adopted for the above multiple radar systems. All radars generate measurements at each sampling instant and report those measurements to the central fusion center (CFC) [27]. It fuses all the acquired measurements in turn and updates the tracks. The fusion architecture is in Figure 1.

where R*_i_* (*i* = 1,2,…*M*) denotes the *q*th radar.

**Figure 2:**
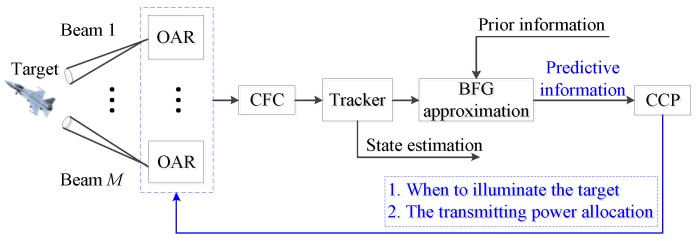
Block diagram of resource management for maneuvering target tracking (MTT).

Hence, in accordance with the fusion architecture, the block diagram of a close-loop intelligent tracking system is given in Figure 2.

The tracked target is initially located at (*x*_T0_, *y*_T0_), and the target is located at (*x*_T*k*_, *y*_T*k*_) at the *k*th sampling interval. For convenience, the (*x*_T*k*_, *y*_T*k*_) is denoted as (*x_k_*, *y_k_*).

### 2.1. Signal Model

It is assumed that the transmitted signal of the *q*th radar to the target at the *k*th sample interval is *s_q_*_,*k*_(*t*):

(1) $$s_{q,k}\left(t\right)=\sqrt{P_{q,k}}S_{q,k}\left(t\right)\exp\left(-j2\pi f_{q}^{c}t\right)$$

where *P_q_*_,*k*_ denotes the transmitted power from the *q*th radar to the target at the *k*th sample interval, and $f_{q}^{c}$ is the carrier frequency. *S_q_*_,*k*_(*t*) denotes the complex envelope of the transmitted signal.

The baseband representation of the echo signal for the *q*th radar at the *k*th sample interval is:

(2) $$r_{q,k}\left(t\right)=h_{q,k}\sqrt{\alpha_{q,k}P_{q,k}}S_{q,k}\left(t-\tau_{q,k}\right)\exp\left(-j2\pi f_{q,k}t\right)+\varpi_{q,k}\left(t\right)$$

where *h_q_*_,*k*_ denotes the target RCS for the *q*th radar, and it is a random variable [25]. Furthermore, *α_q_*_,*k*_∝1/$R_{q,k}^{4}$ denotes the variation of the signal strength due to path loss effects along the path of radar *q*-target-radar *q*. *τ_q_*_,*k*_ means time delay and *f_q_*_,*k*_ is the Doppler frequency. $\;\varpi_{q,k}\left(t\right)$ represents a zero-mean, complex Gaussian white noise with the autocorrelation function $\sigma_{\varpi }^{2}$*δ*(*τ*).

### 2.2. Target Motion Model

As described in previous content, we restrict ourselves to linear jump Markovian systems with an additive Gaussian process noise. Herein, we consider three possible motion models. The three motion models are constant velocity (CV), constant acceleration (CA), and coordinated turn (CT) with a known turn rate, respectively. A larger set of possible motions would be more realistic. Nevertheless, the current set is sufficient to verify the advantage of this algorithm. An overview of other target motion models may be found in References [28,29]. In addition, the dimensionalities of target states for different dynamic models are different, and it may result in a change in the dimensionality of the information matrix, whenever there is a model switch [30]. Hence, to avoid this problem, we set the state vectors with the same dimensionality in different dynamic models. The target state vector consists of the position, velocity, and acceleration in a two-dimensional space. Therefore, the dimensionality of the state vector is six. Details of each motion model used in this paper are given as follows.

#### 2.2.1. Constant Velocity Motion Model

The CV motion model in the *xy* plane is shown below.

(3) $$\xi_{k}=F_{1,k-1}\xi_{k-1}+w_{1,k-1}$$

where $\xi_{k}={\left[x_{k}\;{\dot{x}}_{k}\;0\;y_{k}\;{\dot{y}}_{k}\;0\right]}^{T}$ denotes the state vector, and its dimension is *N*_ξ_ = 6. ${\left[x_{k}\;y_{k}\right]}^{T}$ and ${\left[{\dot{x}}_{k}\;{\dot{y}}_{k}\right]}^{T}$ denote the position and velocity of the target, respectively. The acceleration is [0, 0]^T^. [•]^T^ denotes matrix transposition. **F**_1,*k*−1_ is the 6 × 6 transition matrix.

(4) $$F_{1,k-1}=I_{2}⊗\left[\begin{array}{ccc} 1 & T_{k} & 0\\ 0 & 1 & 0\\ 0 & 0 & 0 \end{array}\right]$$

where ⊗ is the Kronecker operator. **I**_2_ denotes an identity matrix of order 2. *T_k_* is the sampling interval (Let *k* = 0 when the initial time is *t* = 0, and the first sampling interval *T*_1_ is between *k* = 0 and *k* = 1. Thereby, the *k*th sampling interval between epochs *k*−1 and *k* is denoted as *T_k_* in (4). The expression has the same meaning in the remaining paper).

**w**_1,*k*−1_ represents a zero-mean, complex Gaussian white noise, and its covariance is shown below.

(5) $$Q_{1,k-1}=\sigma_{1}•I_{2}⊗\left[\begin{array}{ccc} \frac{1}{3}T_{k}^{3} & \frac{1}{2}T_{k}^{2} & 0\\ \frac{1}{2}T_{k}^{2} & T_{k} & 0\\ 0 & 0 & 0 \end{array}\right]$$

where *σ*_1_ is the process noise intensity [31].

#### 2.2.2. Constant Acceleration Motion Model

The CA motion model is prescribed as follows.

(6) $$\xi_{k}=F_{2,k-1}\xi_{k-1}+w_{2,k-1}$$

where $\xi_{k}={\left[x_{k}\;{\dot{x}}_{k}\;{\ddot{x}}_{k}\;y_{k}\;{\dot{y}}_{k}\;{\ddot{y}}_{k}\right]}^{T}$ denotes the state vector. ${\left[{\ddot{x}}_{k}\;{\ddot{y}}_{k}\right]}^{T}$ denotes the constant acceleration. **F**_2,*k*−1_ is the 6 × 6 transition matrix.

(7) $$F_{2,k-1}=I_{2}⊗\left[\begin{array}{ccc} 1 & T_{k} & \frac{1}{2}T_{k}^{2}\\ 0 & 1 & T_{k}\\ 0 & 0 & 1 \end{array}\right]$$

**w**_2,*k*−1_ is a zero-mean, complex Gaussian white noise with the covariance.

(8) $$Q_{2,k-1}=\sigma_{2}•I_{2}⊗\left[\begin{array}{ccc} \frac{1}{20}T_{k}^{5} & \frac{1}{8}T_{k}^{4} & \frac{1}{6}T_{k}^{3}\\ \frac{1}{8}T_{k}^{4} & \frac{1}{3}T_{k}^{3} & \frac{1}{2}T_{k}^{2}\\ \frac{1}{6}T_{k}^{3} & \frac{1}{2}T_{k}^{2} & T_{k} \end{array}\right]$$

where *σ*_2_ is the process noise intensity of CA [31].

#### 2.2.3. Coordinated Turn Motion Model

In this scenario, we assume that the angular turn rate *Ω* is known and constant. Hence, the target motion model of CT remains linear. Then, the CT motion model is expressed as:

(9) $$\xi_{k}=F_{3,k-1}\xi_{k-1}+w_{3,k-1}$$

where $\xi_{k}={\left[x_{k}\;{\dot{x}}_{k}\;0\;y_{k}\;{\dot{y}}_{k}\;0\right]}^{T}$ denotes the state vector. **F**_3,*k*−1_ is the 6 × 6 transition matrix.

(10) $$F_{3,k-1}=\left[\begin{array}{cccccc} 1 & \frac{\sin\left(\Omega T_{k}\right)}{\Omega } & 0 & 0 & \frac{\cos\left(\Omega T_{k}\right)-1}{\Omega } & 0\\ 0 & \cos\left(\Omega T_{k}\right) & 0 & 0 & -\sin\left(\Omega T_{k}\right) & 0\\ 0 & 0 & 0 & 0 & 0 & 0\\ 0 & \frac{1-\cos\left(\Omega T_{k}\right)}{\Omega } & 0 & 1 & \frac{\sin\left(\Omega T_{k}\right)}{\Omega } & 0\\ 0 & \sin\left(\Omega T_{k}\right) & 0 & 0 & \cos\left(\Omega T_{k}\right) & 0\\ 0 & 0 & 0 & 0 & 0 & 0 \end{array}\right]$$

where *Ω* is the angular turn rate, and **w**_3,*k*−1_ is a zero-mean, complex Gaussian white noise with the covariance given by the equation below.

(11) $$Q_{3,k-1}=\sigma_{3}•I_{2}⊗\left[\begin{array}{ccc} \frac{1}{4}T_{k}^{4} & \frac{1}{3}T_{k}^{3} & 0\\ \frac{1}{3}T_{k}^{3} & \frac{1}{2}T_{k}^{2} & 0\\ 0 & 0 & 0 \end{array}\right]$$

where *σ*_3_ is the process noise intensity of CT [31].

### 2.3. Measurement Model

The measurement equation does not play a role in the BFG approximation. Hence, although the BFG approximation is restricted to linear switching dynamic models, it places no restriction on the measurement equation, which can be nonlinear.

The received signal from the target is an attenuated version of the transmitted signal. The range, azimuth, and Doppler frequency can be extracted from the received signal. The nonlinear measurement equation of the *q*th radar can be written by the equation below.

(12) $$z_{q,k}=h_{q,k}\left(\xi_{k}\right)+u_{q,k}$$

where

(13) $$h_{q,k}\left(\xi_{k}\right)={\left(R_{q,k},\theta_{q,k},f_{q,k}\right)}^{T}$$

with

(14) $$\left\{ \begin{array}{l} R_{q.k}=\sqrt{{\left(x_{k}-x_{q}\right)}^{2}+{\left(y_{k}-y_{q}\right)}^{2}}\\ \theta_{q,k}=\arctan\left[\left(y_{k}-y_{q}\right)/\left(x_{k}-x_{q}\right)\right]\\ f_{q,k}=-\frac{2}{\lambda_{q}}\left({\dot{x}}_{k},{\dot{y}}_{k}\right)\cdot {\left(x_{k}-x_{q},y_{k}-y_{q}\right)}^{T}/R_{q.k} \end{array}\right.$$

corresponding to range, azimuth, and Doppler shift. *λ_q_* is the carrier wavelength of the *q*th radar. The measurement error **u***_q_*_,*k*_ is a zero-mean Gaussian white noise with the covariance.

(15) $$\Sigma_{q,k}=\mathrm{blkdiag}\left(\sigma_{R_{q,k}}^{2}\;\sigma_{\theta_{q,k}}^{2}\;\sigma_{f_{q,k}}^{2}\right)$$

where $\sigma_{R_{q,k}}^{2}$, $\sigma_{\theta_{q,k}}^{2}$, and $\sigma_{f_{q,k}}^{2}$ are the BCRLBs of the mean square error (MSE) of the range, azimuth, and Doppler shift at high signal-to-noise ratio (SNR). The BCRLBs for $\sigma_{R_{q,k}}^{2}$ and $\sigma_{f_{q,k}}^{2}$ are given in Chapter 10 of Reference [31], and the BCRLB for $\sigma_{\theta_{q,k}}^{2}$ which follows from Reference [32]. The BCRLBs can be described as follows.

(16) $$\left\{ \begin{array}{l} \sigma_{R_{q,k}}^{2}=c^{2}/\left[32\pi^{2}\cdot SNR_{q,k}\cdot {\left(B_{q,k}\right)}^{2}\right]\\ \sigma_{\theta_{q,k}}^{2}=3{\left(B_{\mathrm{NN}}\right)}^{2}/\left(8\pi^{2}\cdot SNR_{q,k}\right)\\ \sigma_{f_{q,k}}^{2}=3/\left[8\pi^{2}\cdot SNR_{q,k}\cdot {\left(T_{q,k}\right)}^{2}\right] \end{array}\right.$$

where *B_q,k_* and *T_q_*_,*k*_ are the effective bandwidth and effective time duration [31], respectively. *B*_NN_ is the null-to-null beam width of the receiver antenna. c is the speed of light. *SNR_q,k_* is the SNR denoted as follows [33].

(17) $$SNR_{q,k}=\frac{\alpha_{q,k}{\left|h_{q,k}\right|}^{2}P_{q,k}}{\sigma_{\varpi }^{2}}\propto \frac{{\left|h_{q,k}\right|}^{2}P_{q,k}}{\sigma_{\varpi }^{2}R_{q,k}^{4}}$$

## 3. Resource Management Model for Maneuvering Target Tracking

${\hat{\xi }}_{k}$ is an estimation of the target state $\xi_{k}$. Hence, ${\hat{\xi }}_{k}$ is a function of the measurement value **Z***_k_*, which is represented as follows.

(18) $$Z_{k}={\left\{z_{q,k}\right\}}_{q=1}^{M}$$

The performance of any estimator ${\hat{\xi }}_{k}$ is measured by the MSE matrix. In the Bayesian estimation, the MSE can be bounded by the BCRLB $C_{\mathrm{BCRLB}}^{k}$ (defined to be the inverse of BIM **J**(**ξ***_k_*)) in estimating the random vector $\xi_{k}$, under suitable regularity conditions. For the target with a single motion model, the MSE of any estimator cannot be less than the BCRLB $C_{\mathrm{BCRLB}}^{k}$ [17].

(19) $$E_{\xi_{k},Z_{k}}\left[({\hat{\xi }}_{k}(Z_{k})-\xi_{k}\right){({\hat{\xi }}_{k}(Z_{k})-\xi_{k})}^{T}]\geq C_{\mathrm{BCRLB}}^{k}(\xi_{k})=J^{-1}(\xi_{k})$$

where $E_{\xi_{k},Z_{k}}\left[\cdot \right]$ denotes the expectation over $\xi_{k}$ and **Z***_k_*

The BIM $J(\xi_{k})$ can be described as:

(20) $$J\left(\xi_{k}\right)=E_{\xi_{k},Z_{k}}\left[\left(\frac{\partial \ln p\left(Z_{k},\xi_{k}\right)}{\partial \xi_{k}}\right){\left(\frac{\partial \ln p\left(Z_{k},\xi_{k}\right)}{\partial \xi_{k}}\right)}^{T}\right]$$

The joint PDF *p*(**Z***_k_*,**ξ***_k_*) can be factorized as the product of PDF *p*(**ξ***_k_*) and conditional PDF *p*(**Z***_k_*|**ξ***_k_*).

(21) $$p\left(Z_{k},\xi_{k}\right)=p\left(\xi_{k}\right)p\left(Z_{k}|\xi_{k}\right)$$

Hence, the BIM **J**(**ξ***_k_*) can be divided into a prior information matrix **J**_P_(**ξ***_k_*) and a data information matrix **J**_D_(**ξ***_k_*) [17].

(22) $$J\left(\xi_{k}\right)=J_{P}\left(\xi_{k}\right)+J_{D}\left(\xi_{k}\right)$$

The prior information matrix $J_{P}\left(\xi_{k}\right)$ is determined by the motion equation of targets, and the transmitted power has no impact on it. On the contrary, the data information matrix $J_{D}\left(\xi_{k}\right)$ is affected by the transmitted power allocated. The larger the transmitted power is, the larger the $J_{D}\left(\xi_{k}\right)$ is.

### 3.1. Best-Fitting Gaussian Approximation

To calculate the BIM for linear jump Markovian systems with additive Gaussian process noise through Equation (22), we need to express the dynamics of the system.

(23) $$\xi_{k}=F_{k-1}^{r_{k}}\xi_{k-1}+w_{k-1}^{r_{k}},\;w_{k-1}^{r_{k}}\sim N\left(0,Q_{p,k-1}^{r_{k}}\right)$$

with the BFG approximation:

(24) $$\xi_{k}\approx \Phi_{k-1}\xi_{k-1}+W_{k-1},\;W_{k-1}\sim N(0,Q_{p,k-1})$$

where *r_k_* (*r_k_*= 1, 2, …, *N*_m_ and *N*_m_ is the number of the target motion models) specifies the target motion model in effect during the time interval between sampling time indexes *k*−1 and *k*. **Φ***_k_*_−1_, **W***_k_*_−1_, and **Q**_p,*k*−1_ are the transition matrix, process noise vector, and covariance matrix of the BFG approximation equation, respectively. For convenient representation in the following process of derivation, the notation **F***_i_*_,*k*_ and **Q***_i_*_,*k*_ in Section 2.2 (*i* = 1, 2, …, *N*_m_) are changed to $F_{k-1}^{r_{k}}$ and $Q_{p,k-1}^{r_{k}}$. is an additive Gaussian process noise with $W_{k-1}^{r_{k}}\sim N\left(0,Q_{p,k-1}^{r_{k}}\right)$.

At each sampling interval, the regime-dependent motion model (given by Equation (23) and known as “model 1”) is replaced with a single BFG distribution (denoted by Equation (24) and referred to as “model 2”). **Φ***_k_*_−1_ and **Q**_p,*k*−1_ (**Q**_p,*k*−1_ ≥ 0) are calculated to satisfy the requirements that the distribution of $\xi_{k}$ has the same mean and covariance under different models at each stage.

(25) $$E[\xi_{k}|\mathrm{model}\;1]=E[\xi_{k}|\mathrm{model}\;2]$$

(26) $$\mathrm{Cov}\left[\xi_{k}|\mathrm{model}\;1\right]=\mathrm{Cov}\left[\xi_{k}|\mathrm{model}\;2\right]$$

where E[•] and Cov[•] denote the expectation operator and covariance operator, respectively.

The computation procurement of **Φ**_*k*−1_ is given in Appendix A. Then, with the aid of **Φ**_*k*−1_, we obtain **Q**_p,*k*−1_ by the detailed derivation in Appendix B.

(27) $$\varepsilon_{k}≜E[\xi_{k}|\mathrm{model}\;1]=E[\xi_{k}|\mathrm{model}\;2]$$

(28) $${𝓒}_{k}≜\mathrm{Cov}\left[\xi_{k}|\mathrm{model}\;1\right]=\mathrm{Cov}\left[\xi_{k}|\mathrm{model}\;2\right]$$

It is assumed that the initial distribution of state vector **ξ** is Gaussian with mean ${\bar{\xi }}_{0}$ and covariance $J_{P}^{-1}(\xi_{0})$. Generally speaking, initially, the target is in a CV motion. Hence, the **ε**_1_ and 𝓒_1_ can be calculated easily. The transition probability is *p_ij_* ≜ Pr(*r_k_*= *j*|*r_k_*_−1_ = *i*) (*i*, *j* = 1, 2, …, *N*_m_). To proceed, the recursive computation procedure for **Φ***_k_*_−1_ and **Q**_p,*k*−1_ is given as follow:

Step 1. Let *k* = 1, and initialize **ε***_k_*_−1_, 𝓒_k−1_, and the mode probabilities *p_k_*_−1_(*r*), *r* = 1, 2, …, *N*_m_

Step 2. Calculate the mode probabilities.

(29) $$p_{k}(r)=\sum_{j=1}^{N_{m}}p_{jr}p_{k-1}(j)$$

Step 3. By derivation, obtain **Φ***_k_*_−1_ of BFG distribution as (see Appendix A):

(30) $$\Phi_{k-1}=\left[\sum_{r=1}^{N_{m}}F_{k-1}^{r}p_{k}\left(r\right)\right]$$

Step 4. With the aid of **Φ***_k_*_−1_, derive the covariance matrix 𝓒*_k_*.

(31) $${𝓒}_{k}=\sum_{r=1}^{N_{m}}p_{k}\left(r\right)\left[F_{k-1}^{r}\left({𝓒}_{k-1}+\varepsilon_{k-1}\varepsilon_{k-1}^{T}\right){\left(F_{k-1}^{r}\right)}^{T}+Q_{k-1}^{r}\right]-\Phi_{k-1}\varepsilon_{k-1}\varepsilon_{k-1}^{T}\Phi_{k-1}^{T}$$

Step 5. Calculate **Q**_p,*k*−1_ as follows:

(32) $$Q_{p,k-1}={𝓒}_{k}-\Phi_{k-1}{𝓒}_{k-1}\Phi_{k-1}^{T}$$

Step 6. Update the mean of the state vector.

(33) $$\varepsilon_{k}=\Phi_{k-1}\varepsilon_{k-1}$$

Step 7. Let *k* = *k* + 1, and go to Step 2.

### 3.2. Prior Information ***J***_*P*_(***ξ***_k_)

The prior information matrix **J**_P_(**ξ***_k_*) can be formulated by the equation below [20].

(34) $$J_{P}\left(\xi_{k}\right)=E_{\xi_{k}}\left[\left(\frac{\partial \ln p\left(\xi_{k}\right)}{\partial \xi_{k}}\right){\left(\frac{\partial \ln p\left(\xi_{k}\right)}{\partial \xi_{k}}\right)}^{T}\right]=D_{k-1}^{22}-D_{k-1}^{21}{\left[J\left(\xi_{k-1}\right)+D_{k-1}^{11}\right]}^{-1}D_{k-1}^{12}$$

According the calculated **Φ***_k_*_−1_ and **Q**_p,*k*−1_ by the linear BFG approximation, the $D_{k-1}^{11}$, $D_{k-1}^{12}$, and $D_{k-1}^{22}$ are deterministic and the expectation operator can be dropped out.

(35) $$\left\{ \begin{array}{l} D_{k-1}^{11}=\Phi_{k-1}^{T}Q_{p,k-1}\Phi_{k-1}\\ D_{k-1}^{12}=-\Phi_{k-1}^{T}{\left[Q_{p,k-1}\right]}^{-1}\\ D_{k-1}^{22}={\left[Q_{p,k-1}\right]}^{-1} \end{array}\right.$$

Substituting (35) into (34), we can derive the prior information matrix.

(36) $$J_{P}\left(\xi_{k}\right)={\left[Q_{p,k-1}+\Phi_{k-1}J^{-1}\left(\xi_{k-1}\right)\Phi_{k-1}^{T}\right]}^{-1}$$

### 3.3. Data Information ***J**_D_*(***ξ***_k_)

On the other hand, the data information matrix **J**_D_(**ξ***_k_*) can be expressed by the equation below [20].

(37) $$J_{D}\left(\xi_{k}\right)=E_{\xi_{k},Z_{k}}\left[\left(\frac{\partial \ln p\left(Z_{k}|\xi_{k}\right)}{\partial \xi_{k}}\right){\left(\frac{\partial \ln p\left(Z_{k}|\xi_{k}\right)}{\partial \xi_{k}}\right)}^{T}\right]$$

Since the measurements from different radars are independent from each other, **J**_D_(**ξ**_k_) can be written as follows.

(38) $$\begin{array}{l} J_{D}\left(\xi_{k}\right)=\sum_{q=1}^{M}J_{D}^{q}\left(\xi_{k}\right)\\ =\sum_{q=1}^{M}E_{\xi_{k},z_{q,k}}\left[\left(\frac{\partial \ln p\left(z_{q,k}|\xi_{k}\right)}{\partial \xi_{k}}\right){\left(\frac{\partial \ln p\left(z_{q,k}|\xi_{k}\right)}{\partial \xi_{k}}\right)}^{T}\right]\\ =\sum_{q=1}^{M}E_{\xi_{k}}\left[H_{q,k}^{T}\Sigma_{q,k}^{-1}H_{q,k}\right] \end{array}$$

where $H_{q,k}={\left[\partial h_{q}^{T}\left(\xi_{k}\right)/\partial \xi_{k}\right]}^{T}$.

Substituting Equations (36) and (38) into Equation (22), we can now calculate the BIM **J**(**ξ**_k_) by using the Riccati-like recursion [20].

(39) $$J\left(\xi_{k}\right)={\left[Q_{p,k-1}+\Phi_{k-1}J^{-1}\left(\xi_{k-1}\right)\Phi_{k-1}^{T}\right]}^{-1}+\sum_{q=1}^{M}E_{\xi_{k}}\left[H_{q,k}^{T}\Sigma_{q,k}^{-1}H_{q,k}\right]$$

In practice, the expected value in Equation (39) may be evaluated using Monte Carlo techniques. To shorten the computation time, the predictive BIM is approximated as follows [34].

(40) $$J\left(\xi_{k}\right)={\left[Q_{p,k-1}+\Phi_{k-1}J^{-1}\left(\xi_{k-1}\right)\Phi_{k-1}^{T}\right]}^{-1}+{\left(\sum_{q=1}^{M}H_{q,k}^{T}\Sigma_{q,k}^{-1}H_{q,k}\right)|}_{\xi_{k|k-1}}$$

where **ξ***_k_*_|*k*−1_ denotes the predicted target state for the case of zero process noise.

### 3.4. Predictive Bayesian Cramér-Rao lower bound (BCRLB-like)

The key feature of the resource allocation algorithm is the predictability. The predictive target tracking performance gives us the ability to make decisions in advance based on current knowledge. In the maneuvering target tracking, **Φ***_k_*_−1_ and **Q**_p,*k*−1_ are updated through Equations (30) and (32) before each revisit time. They are both functions of the sampling interval *T_k_*. Hence, the mathematical symbol of the prior information matrix can be modified from **J**_P_(**ξ***_k_*) to **J**_P_(*T_k_*)|**_ξ_***_k_* (**J**_P_(*T_k_*) has nothing to do with **ξ***_k_*. Nevertheless, to keep the consistency of the format, it is denoted as $J_{P}\left(T_{k}\right)|{\;}_{\xi_{k}}$ here). The data information matrix is affected by the transmitted power of the radars and the target RCS. The larger the transmitted power **P***_k_* and the RCS **h***_k_* are, the larger the data information matrix is. Consequently, the data information matrix can be rewritten as **J**_D_(**P***_k_*,**h***_k_*)|**_ξ_***_k_*. Considering the updated BIM **J**(**ξ**_*k*−1_), sampling interval *T_k_*, transmitted power **P***_k_* and RCS **h***_k_*, we can calculate the predictive BIM.

(41) $$\begin{array}{cc} {J\left(T_{k},P_{k},h_{k}\right)|}_{\xi_{k}} & ={J_{P}\left(T_{k}\right)|}_{\xi_{k}}+{J_{D}\left(P_{k},h_{k}\right)|}_{\xi_{k}}\\ & ={\left[Q_{p,k-1}\left(T_{k}\right)+\Phi_{k-1}\left(T_{k}\right)J^{-1}\left(\xi_{k-1}\right)\Phi_{k-1}^{T}\left(T_{k}\right)\right]}^{-1}+{\left[\sum_{q=1}^{M}H_{q,k}^{T}\Sigma_{q,k}^{-1}\left(P_{q,k},h_{k}^{q}\right)H_{q,k}\right]|}_{\xi_{k|k-1}} \end{array}$$

where the meaning of **ξ***_k_*_|*k*−1_ refers to Equation (40).

The predictive target tracking performance can be calculated as the inverse of BIM. However, because of the BFG approximation technique involved, the predictive target tracking performance no longer provides a lower boundary on error performance. Hence, the predictive target tracking performance in this paper is named BCRLB-like instead of BCRLB.

(42) $${C_{\mathrm{BCRLB}-\mathrm{like}}\left(T_{k},P_{k},h_{k}\right)|}_{\xi_{k}}={\left[{J\left(T_{k},P_{k},h_{k}\right)|}_{\xi_{k}}\right]}^{-1}$$

The diagonal elements of BCRLB-like provide a referenced boundary on the variances of the estimation of the target’s hybrid state. It is sufficient for us to utilize the tracking BCRLB-like factor of the illuminated target as a criterion for power allocation.

(43) $$F\left(T_{k},P_{k},h_{k}\right)=\sqrt{\mathrm{trace}\left[{C_{\mathrm{BCRLB}-\mathrm{like}}\left(T_{k},P_{k},h_{k}\right)|}_{\xi_{k}}\right]}$$

where Equation (43) denotes the integrated tracking performance of multiple radars at the *k*th sampling instant.

Having the tracking BCRLB-like *F*(*T_k_*,**P***_k_*,**h***_k_*), we can allocate the power resource reasonably while the beams illuminate the target. However, due to the scarce radar resource, the radars do not have to illuminate the target continually. The beams only need to be transmitted when the tracking error is larger than a given tracking error threshold. As shown in Equations (22) and (36), the prior information matrix $J_{P}\left(T_{k}\right)$ is only related to a target motion equation. Therefore, the inverse of $J_{P}\left(T_{k}\right)$ is utilized to determine when to transmit the beams.

(44) $$C_{\mathrm{CRLB}-\mathrm{like}}^{P}\left(T_{k}\right)={\left[{J_{P}\left(T_{k}\right)|}_{\xi_{k}}\right]}^{-1}$$

To accomplish an adaptive sampling interval, the prior CRLB-like is compared with the upper boundary of the given tracking error threshold. It can be used as a criterion to determine the optimal sampling period. The representation of the prior CRLB-like factor is given as follows.

(45) $$F_{P}\left(T_{k}\right)=\sqrt{\mathrm{trace}\left[C_{\mathrm{CRLB}-\mathrm{like}}^{P}\left(T_{k}\right)\right]}$$

### 3.5. Modeling of Chance-Constraint Programming (CCP)

In accordance with Equation (16), it can be seen that the target tracking accuracy is impacted by several parameters. The decision variables involved in this paper are the sampling interval *T_k_* and the transmit power **P***_k_*. The random vector is the RCS **h***_k_*. For the predetermined tracking error range at each sampling instant, the aim of our work is to minimize the transmitted power by optimally allocating the limited time and power resources.

(46) $$\begin{array}{cc} \min & 1_{M}^{T}P_{k}\\ s.t. & \\ & P_{q,k}\geq P_{\min}\;q=1,2,\cdots ,M\\ & 1_{M}^{T}P_{k}\leq P_{\mathrm{total}}\\ & F\left(T_{k},P_{k},h_{k}\right)\leq \eta_{1,k}\;\mathrm{if}\;F_{P}\left(T_{k}\right)\geq \eta_{2,k} \end{array}$$

where $1_{M}^{T}$ = [1,1,…,1]_1×*M*_. *P*_min_ is the lower boundary of the transmitted power of each beam. *P*_total_ is the available total power. *η*_1,*k*_ and *η*_2,*k*_ are the lower boundary and upper boundary of the tracking error range, respectively. The adaptive sampling interval is included in the resource management model. If the prior CRLB-like *F*_P_(*T_k_*) of the next sampling instant is greater than the given tracking error threshold *η*_2,*k*_, *T_k_* is set as the sampling interval and the beams are transmitted to track the target.

In Equation (46), the model can make sure that the tasks can be accomplished by as many as possible conditions on the given tracking error threshold.

In practice, the target RCS is related to the identification, attitude, and position of the target, and it is affected by an aspect angle, wavelength, and polarization, etc. [25], i.e., the target RCS is unknown and uncertain. Consequently, the target RCS is considered as a random variable in this paper. Then the deterministic resource allocation model cannot show the characteristic of the target well. In view of the above situation, the stochastic CCP of the resource management is introduced [26]. According to Equation (46), the resource management model can be reformulated as:

(47) $$\begin{array}{cc} \min & 1_{M}^{T}P_{k}\\ s.t. & \\ & P_{q,k}\geq P_{\min}\;q=1,2,\cdots ,M\\ & 1_{Q}^{T}P_{k}\leq P_{\mathrm{total}}\\ & \mathrm{Pr}\left\{F\left(T_{k},P_{k},h_{k}\right)\leq \eta_{1,k}\right\}\geq \alpha \;\mathrm{if}\;F_{P}\left(T_{k}\right)\geq \eta_{2,k} \end{array}$$

where Pr{•} is a probability measure operator. *α* is a confidence level.

## 4. Resource Allocation Processing Procedure

### 4.1. Basic of the Technique

As a type of stochastic programming pioneered by Charnes and Cooper [35], the CCP offers a powerful means of modeling stochastic decision systems with the assumption that the stochastic constraints will hold at least *α* of time, where *α* is referred to as the confidence level provided as an appropriate safety margin by the decision-maker. It is convenient and general to deal with them by stochastic simulations. Hence, Liu [26] integrates the stochastic simulation and GA to produce a hybrid intelligent optimization algorithm (HIOA) for solving stochastic programming models.

In recent years, some new filtering algorithms have emerged one after another. The particle filter has the highest filtering accuracy [36,37]. However, since it is based on Monte Carlo methods, the calculation time of the particle filter is longer. The shadowing filter offers a robust methodology to position and track a moving target from limited positional information [38,39]. Nevertheless, the target information of this paper is not incomplete, or else the resource allocation process cannot be performed successfully. Under the condition that the filtering accuracy satisfies the calculation requirement of this paper, the unscented Kalman Filter (UKF) has a faster filtering speed [40]. In addition, it can also solve the filtering problems whose target information is complete. For the previously mentioned reasons, the UKF is selected.

In addition, since the dynamics of the linear jump Markovian system are expressed by the BFG approximation, the radars can track the maneuvering target by a UKF. On the other hand, we also employ the interacting multiple model UKF (IMM-UKF) to track the maneuvering target [40,41,42]. Thus, the tracking performance of BFG-UKF can be compared with the tracking performance of IMM-UKF to verify the efficiency of the BFG approximation.

The resource allocation processing procedure can be detailed as follows.

### 4.2. Stochastic Simulation

For the randomness of the target RCS, the stochastic simulation is used to calculate the stochastic CCP [26]. According to the expert experience and historical measurement data, it is assumed that there exists *Nh_q,k_* (*Nh_q,k_* is the number of measurements relative to the *q*th radar.) measurements before sampling, and each measurement is denoted as $h_{i,k}^{q}$ (*i* = 1, 2, …, *Nh_q,k_*). Let *N_H_*= *Nh*_1*,k*_×*Nh*_2*,k*_×…×*Nh_q,k_*. When the resource will be pre-allocated at the *k*th sampling time, we can select **h***_j_*_,*k*_ (*j* = 1, 2, …, *N_H_*) from the measurement set of RCS to produce *F*(*T_k_*,**P***_k_*,**h***_j_*_,*k*_). Let *N′* denote the number of occasions on which *F*(*T_k_*,**P***_k_*,**h***_j_*_,*k*_) ≤ *η*_1,*k*_ (i.e., the number of random vectors satisfy the system of inequalities). Let us define the following.

(48) $$h_{F}\left(T_{k},P_{k},h_{j,k}\right)=\left\{ \begin{array}{l} 1,\;\mathrm{if}\;F\left(T_{k},P_{k},h_{j,k}\right)\leq \eta_{1,k}\\ 0,\;\mathrm{otherwise} \end{array}\right.$$

It follows from the strong law of large numbers that:

(49) $$\frac{N^{\prime }}{N_{H}}=\frac{\sum_{i=1}^{N_{H}}h_{F}\left(T_{k},P_{k},h_{j,k}\right)}{N_{H}}$$

This converges a.s. to Pr{*F*(*T_k_*,**P***_k_*,**h***_k_*)≤*η*_1,*k*_}. Thus, the probability measure can be estimated by *N*′/*N_H_*, which provided that *N_H_* is sufficiently large. The solving steps is illustrated in Table 1.

### 4.3. Hybrid Intelligent Optimization Algorithm

In Equation (47), if *F*_P_(*T_k_*)≥*η*_2,*k*_, we will solve the stochastic CCP model to obtain the optimal power allocation **P***_k_*_,opt_. Hence, the optimal value *T_k_*_,opt_ has been calculated before solving the stochastic CCP model. Then, we just need to solve the stochastic CCP model to obtain the optimal power allocation **P***_k_*_,opt_. The stochastic simulation is introduced and embedded into GA to constitute HIOA for solving the stochastic CCP model [26]. The steps are described in Table 2.

### 4.4. Target State Estimation

For a maneuvering target, the key to successful target tracking lies in the effective extraction of useful information about the target’s state from observations. Furthermore, a good model of the target will facilitate this information extraction process to a great extent. In this paper, the dynamics of the linear jump Markov system can be replaced by the BFG approximation with a Gaussian distribution. The transition matrix and process noise of the BFG equation need to be updated before each sampling instant. Therefore, the maneuvering target state can be estimated by BFG-UKF (Herein, for the sake of clarity, the UKF including the BFG approximation is renamed as BFG-UKF). In addition, the IMM-UKF is a wildly accepted state estimation method for MTT [41,42]. Hence, we compare the tracking effects between BFG-UKF and IMM-UKF. The target state estimation processes are detailed in the following, respectively.

#### 4.4.1. Process of BFG-UKF

Step 1. Initialization: let *k* = 1, and initialize *T_k_*_,opt_ = Δ*T* (Δ*T* denotes a short enough time slot during which the change of the target tracking error can be neglected), **P***_k_*_,opt_ = **P**_0_ (**P**_0_ denotes equal power allocation), the mode probability *p_k_*_−1_(*i*), the transition probability *p_ij_*, the state **ξ***_k_*_−1|*k*−1_, and the covariance **C***_k_*_−1|*k*−1_ = **$J_{P}^{-1}$**(**ξ***_k_*_−1|*k*−1_).

Step 2. BFG approximation: through the BFG approximation in Section 3.1, determine the mode probability *p_k_*(*i*), and then calculate **Φ***_k_*_−1_ and **Q**_p,*k*−1_ (the detailed calculation process is given in Section 3.1).

Step 3. Prediction procedure: predict the target state and covariance.

(50) $$\left\{ \begin{array}{l} \xi_{k|k-1}=\Phi_{k-1}\xi_{k-1|k-1}\\ C_{k|k-1}=\Phi_{k-1}C_{k-1|k-1}\Phi_{k-1}^{T}+Q_{p,k-1} \end{array}\right.$$

To implement the sequential updating scheme of centralized tracking of multiple radars [27,43], the target state and covariance are rewritten as:

(51) $$\left\{ \begin{array}{l} \xi_{k|k}^{0}=\xi_{k|k-1}\\ C_{k|k}^{0}=C_{k|k-1} \end{array}\right.$$

Step 4. Calculation of sigma sampling points and their weights. For *M* radars, the measurement from the most accurate radar should be updated first so as to reduce subsequent linearization errors. Let *q* = 1, and we start the recursion from the predicted state and covariance denoted by the equations below.

(52) $$\left\{ \begin{array}{l} \xi_{k|k-1}^{q}=\xi_{k|k}^{q-1}\\ C_{k|k-1}^{q}=C_{k|k}^{q-1} \end{array}\right.$$

where *q* = 1, 2, …, *M*. Hence, the (2*N***_ξ_**+1) sigma sampling points $\chi_{l,k|k-1}^{q}$ and their weights *ω_l_*_,*k*_ can be computed in accordance with the following equations.

(53) $$\left\{ \begin{array}{l} \chi_{0,k|k-1}^{q}=\xi_{k|k-1}^{q}\\ \chi_{l,k|k-1}^{q}\\ \;\;\;=\xi_{k|k-1}^{q}+{\left(\sqrt{{\bar{C}}_{k|k-1}^{q}}\right)}_{l}\;\;l=1,2,\cdots ,N_{\xi }\\ \chi_{l,k|k-1}^{q}\\ \;\;\;=\xi_{k|k-1}^{q}-\;{\left(\sqrt{{\bar{C}}_{k|k-1}^{q}}\right)}_{l-N_{\xi }}\;\;l=N_{\xi }+1,N_{\xi }+2,\cdots ,2N_{\xi } \end{array}\right.$$

(54) $$\left\{ \begin{array}{l} \omega_{0,k}=\frac{\varsigma }{N_{\xi }+\varsigma }\\ \omega_{l,k}=\frac{1}{2\left(N_{\xi }+\varsigma \right)}\;\;l=1,2,\cdots ,2N_{\xi } \end{array}\right.$$

where ${\overset{-}{C}}_{k|k-1}^{q}$ = (*N***_ξ_**+*ς*)$C_{k|k-1}^{q}$. *ς* is a scaling parameter. ${\left(\sqrt{•}\right)}_{l}$) is the *l*th row or column of the matrix square root. *N***_ξ_** is the dimensionality of the state vector. *ω_l_*_,*k*_ is the weight that is associated with the *l*th point.

Step 5. Prediction procedure of observations. Transform each sigma point through the measurement equation, and compute the mean $z_{k|k-1}^{q}$, covariance $C_{zz,k}^{q}$, and cross covariance $C_{\xi z,k}^{q}$.

(55) $$\left\{ \begin{array}{l} \zeta_{l,k|k-1}^{q}=h_{q,k}\left(\chi_{l,k|k-1}^{q}\right)\\ z_{k|k-1}^{q}=\sum_{l=0}^{2N_{\xi }}\omega_{l,k}\cdot \zeta_{l,k|k-1}^{q}\\ C_{zz,k}^{q}=\sum_{l=0}^{2N_{\xi }}\omega_{l,k}\Delta z_{l,k|k-1}^{q}{\left[\Delta z_{l,k|k-1}^{q}\right]}^{T}+\Sigma_{q,k}\\ C_{\xi ,k}^{q}=\sum_{l=0}^{2N_{\xi }}\omega_{l,k}\Delta \xi_{l,k|k-1}^{q}{\left[\Delta z_{l,k|k-1}^{q}\right]}^{T} \end{array}\right.$$

where Δ$\xi_{l,k|k-1}^{q}$ = $\chi_{l,k|k-1}^{q}$−$\xi_{k|k-1}^{q}$ and Δ$z_{l,k|k-1}^{q}$ = $\zeta_{l,k|k-1}^{q}$−$z_{k|k-1}^{q}$.

Step 6. Target state updating: calculate the gain $K_{k}^{q}$, and update the state vector $\xi_{k|k}^{q}$ and covariance $C_{k|k}^{q}$.

(56) $$\left\{ \begin{array}{l} K_{k}^{q}=C_{\xi z,k}^{q}\cdot {\left(C_{zz,k}^{q}\right)}^{-1}\\ \xi_{k|k}^{q}=\xi_{k|k-1}^{q}+K_{k}^{q}\cdot \left[z_{q,k}-z_{k|k-1}^{q}\right]\\ C_{k|k}^{q}=C_{k|k-1}^{q}-K_{k}^{q}\cdot C_{zz.k}^{q}\cdot {\left(K_{k}^{q}\right)}^{T} \end{array}\right.$$

where **z***_q_*_,*k*_ is the measurement of the *q*th radar.

Step 7. Let *q* = *q* + 1, and if *q* ≤ *M*, go to Step 4. Otherwise, go to Step 8.

Step 8. Adaptive sampling interval: let *T_k_*_+1_ = 0.

1) Let *T_k_*_+1_ = *T_k_*_+1_+Δ*T*, and through the BFG approximation in Section 3.1, determine the mode probability *p_k+_*_1_(*i*), and then calculate **Φ***_k_*_+1_ and **Q**_p,*k*+1_ (the detailed calculation process is given in Section 3.1).
2) Predict the prior CRLB-like *F*_P_(*T_k_*_+1_) according to Equations (40) and (45).
3) If *F*_P_(*T_k_*_+1_) > *η*_2,*k*+1_ (the upper bound of the error threshold), let *T_k_*_+1,opt_ = *T_k_*_+1_ and go to Step 9. Otherwise, go to Step 1).

Step 9. Power optimal allocation: implement the power allocation algorithm in Table 2 and send the optimal allocation result to the multiple OAR system.

Step 10. Let *k* = *k* + 1, and go to Step 3.

#### 4.4.2. Process of Interacting Multiple Model Unscented Kalman Filter (IMM-UKF)

As we all know, IMM-UKF is an organic combination of IMM and UKF. Then, there exist many common steps between the processes of IMM-UKF and BFG-UKF. Hence, in view of the length of this paper and avoiding redundancy in content, we will simplify the steps that have appeared in the process of BFG-UKF.

Step 1. Initialization: let *k* = 1, and initialize *T_k_*_,opt_ = Δ*T* (Δ*T* denotes a short enough time slot during which the change of the target tracking error can be neglected), **P***_k_*_,opt_ = **P**_0_ (**P**_0_ denotes equal power allocation), the motion model probability *p_k_*_−1_(*i*), the transition probability *p_ij_*, the state **ξ***_k_*_−1|*k*−1_(*i*), and the covariance **C***_k_*_−1|*k*−1_(*i*) = **$J_{P}^{-1}$**(**ξ***_k_*_−1|*k*−1_(*i*)) for a mode-matched filter *i*, where *i*, *j* = 1,2, …, *N*_m_.

Step 2. Interactive input: calculate the mixing probability.

(57) $$p_{k-1|k-1}\left(i|j\right)=p_{ij}p_{k-1}\left(i\right)/p_{k|k-1}\left(j\right)$$

where *p_k_*_|*k*−1_(*j*) is a predicted mode probability.

(58) $$p_{k|k-1}\left(j\right)=\sum_{i=1}^{N_{m}}p_{ij}p_{k-1}\left(i\right)$$

Thus, the mixing state estimation and the mixing covariance are computed interactively as:

(59) $$\left\{ \begin{array}{c} \xi_{k-1|k-1}\left(0j\right)=\sum_{i=1}^{N_{m}}\xi_{k-1|k-1}\left(i\right)p_{k-1|k-1}\left(i|j\right)\\ C_{k-1|k-1}\left(0j\right)=\sum_{i=1}^{N_{m}}p_{k-1|k-1}\left(i|j\right)\{C_{k-1|k-1}\left(i\right)\\ +\left[\xi_{k-1|k-1}\left(i\right)-\xi_{k-1|k-1}\left(0j\right)\right]{\left[\xi_{k-1|k-1}\left(i\right)-\xi_{k-1|k-1}\left(0j\right)\right]}^{T}\} \end{array}\right.$$

Step 3. Filtering of each motion model (*j* = 1, 2, …, *N*_m_): for motion model *j*, the filtering process is the same as the process from Step 3 to Step 7 of BFG-UKF.

Step 4. Mode-probability updating (for *j* = 1, 2, …, *N*_m_): update the model with the following equation.

(60) $$p_{k}\left(j\right)=p_{k|k-1}\left(j\right)\Lambda_{k}\left(j\right)/\sum_{i=1}^{N_{m}}p_{k|k-1}\left(i\right)\Lambda_{k}\left(i\right)$$

where the model likelihood function is:

(61) $$\Lambda_{k}\left(j\right)=\frac{1}{\sqrt{2\pi \left|C_{zz,k}^{M}\left(j\right)\right|}}\cdot \exp\left\{-\frac{1}{2}{\left[\Delta z_{k|k-1}^{M}\left(j\right)\right]}^{T}\left[C_{zz,k}^{M}\left(j\right)\right]\left[\Delta z_{k|k-1}^{M}\left(j\right)\right]\right\}$$

where Δ**z**${}_{k|k-1}^{M}$(*j*) = **z***_M_*_,*k*_ − **z**${}_{k|k-1}^{M}$(*j*).

Step 5. Estimation fusion: fuse the states and covariances of all the mode-matched filters to compute the overall estimate and overall covariance.

(62) $$\left\{ \begin{array}{c} \xi_{k|k}=\sum_{j=1}^{N_{m}}\xi_{k|k}^{M}\left(j\right)p_{k}\left(j\right)\\ C_{k|k}\\ =\sum_{j=1}^{N_{m}}p_{k}\left(j\right)\left\{C_{k|k}^{M}\left(j\right)+\left[\xi_{k|k}^{M}\left(j\right)-\xi_{k|k}\right]{\left[\xi_{k|k}^{M}\left(j\right)-\xi_{k|k}\right]}^{T}\right\} \end{array}\right.$$

Step 6. Adaptive sampling interval: this step is the same as Step 8 of BFG-UKF.

Step 7. Power optimal allocation: this step is the same as Step 9 of BFG-UKF.

Step 8. Let *k* = *k* + 1, and go to Step 2.

## 5. Simulation Results and Analysis

To better illustrate the effectiveness of the proposed resource management scheme, the relevant numerical examples are given in this section. First, we demonstrate the advantage of the adaptive sampling algorithm by comparing it to the fixed sampling algorithm. Then, the total transmitted power is calculated to different confidence levels to account for the impacts of confidence levels on power consumption. Third, by comparing the results of the optimal power allocation and the equal power allocation, the optimal power allocation can not only save the total transmitted power strikingly, but also allocate the power among the distributed radars reasonably. Lastly, the tracking performance of BFG-UKF is compared with the tracking performance of IMM-UKF to demonstrate the validity of the BFG approximation. Now, we give the simulation setup first in the following.

**Figure 3:**
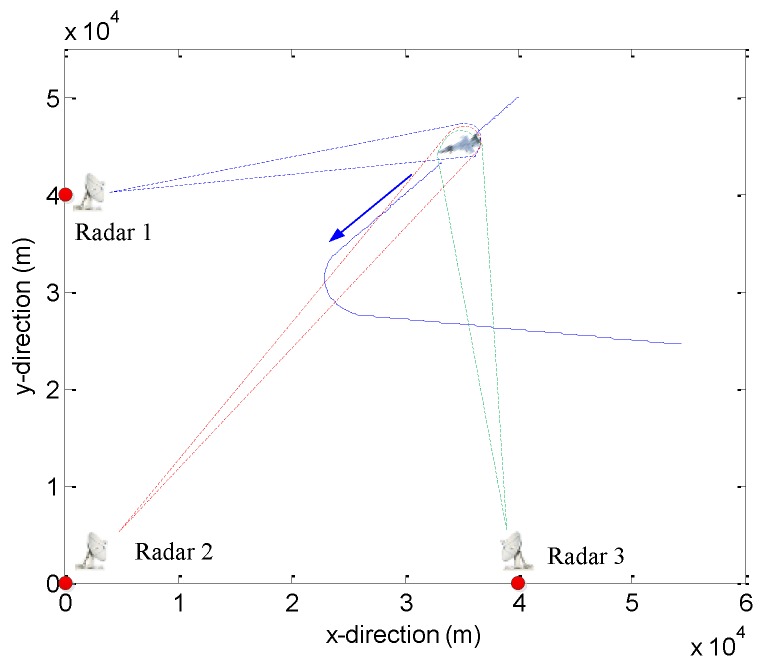
Target tracking scenario.

Suppose that a radar network with *M* = 3 distributed radars is considered. The carrier frequency of each radar is set as 10 GHz, and, thus, the carrier wavelength is *λ_q_*= 0.03 m. The effective bandwidth and effective time duration of each radar beam are set as *B_q_*_,*k*_ = 5 MHz and *T_q_*_,*k*_ = 1 ms, respectively. The time slot of the tracking process is set to Δ*T* = 0.5 s. The number of the coherent pulse is 64. The lower bound of the transmitted power of the *q*th radar is *P_q_*_,min_ = 0.01*P*_total_. The desired tracking error range is [400, 1000] m. The target is initially located at (40, 50) km. From 0 to 50 s, the target first flies with a constant speed of (−200, 200) m/s, and from 51 to 70 s. A CA motion is taken with an acceleration of 10 m/s^2^ in the *x* and *y* coordinate directions, and from 71 to 85 s, it takes a CT motion with a known angular turn rate *Ω* = 0.15 rad/s. Lastly, it keeps moving in a CV motion mode from 86 to 135 s. The maneuvering target trajectory and the deployment of the radars in the tracking scenario is in Figure 3.

To compare the simulation results fairly and conveniently, it is assumed that all the simulation examples have the same target RCS. The total sampling times of the adaptive sampling algorithm cannot be predetermined in the tracking process. Then, we cannot also determine the number of the observed value of RCS. Herein, we give the changing curve of RCS according to the fixed sampling algorithm with the most sampling times.

**Figure 4:**
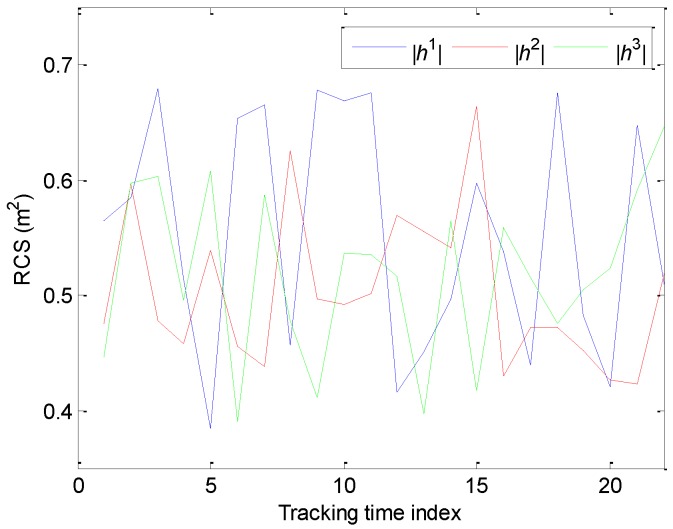
The radar cross section (RCS) of the target relative to different radars.

Where |*h^i^*| is the modulus of target RCS relative to the *i*th radar in Figure 4.

To proceed, the simulation results and corresponding analysis are presented in the following subsections. In the first three subsections, we all adopt the BFG-UKF for target state estimation. In the last subsection, we compare the tracking effects between BFG-UKF and IMM-UKF.

### 5.1. Adaptive Sampling Interval

**Figure 5:**
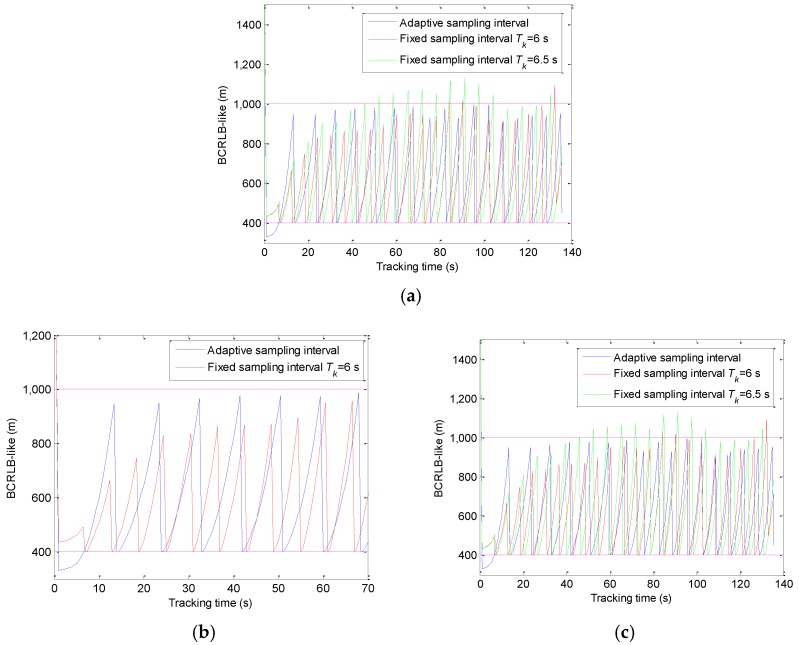
The comparison between the adaptive sampling interval and the fixed sampling interval with different tracking time (**a**) 0–135 s; (**b**) 0–70 s; (**c**) 70–135 s.

This subsection supports the evaluation of the adaptive sampling algorithm. According to the previous analysis in Section 3.4, the adaptive sampling happens before the power allocation, and we predict the tracking error of the target by *F*_P_(*T_k_*) to determine the sampling interval. The visual simulation results are shown in Figure 5.

The simulation results of the adaptive sampling algorithm and the fixed sampling algorithm are compared in Figure 5. In Figure 5a, besides the adaptive sampling algorithm, we also give two simulation results of the fixed sampling algorithm: *T_k_*= 6 s and *T_k_*= 6.5 s. When *T_k_*= 6.5 s, it can be seen from Figure 5 that the prior CRLB-like value does not satisfy the error threshold constraint in many moments. When *T_k_*= 6 s, the prior CRLB-like basically meets with the error threshold constraint during the whole tracking process. Hence, the fixed sampling interval *T_k_*= 6 s is compared with the adaptive sampling interval detailed in Figure 5b,c. During the whole tracking process, the adaptive sampling times 18 is significantly less than the fixed sampling times 22. In Figure 5b, the adaptive sampling interval is approximately 1.5 to 2 times of the fixed sampling interval *T_k_*= 6 s. The motion state is simple and the velocity is low from 0 to 70 s, so the target tracking error increases slowly, and then the radar system will select a long sampling interval intelligently. In Figure 5c, with the increase of maneuverability and velocity of the target, the length of the adaptive sampling interval is reduced to the length of the fixed sampling interval.

### 5.2. Optimal Allocation of Power

In this part, when the adaptive sampling algorithm is used for determining the sampling interval, we contrastively analyze the results of optimal power allocation and equal power allocation conditioned on the desired tracking error threshold. By restricting generality, the confidence level is set as *α* = 0.95 in this simulation.

**Figure 6:**
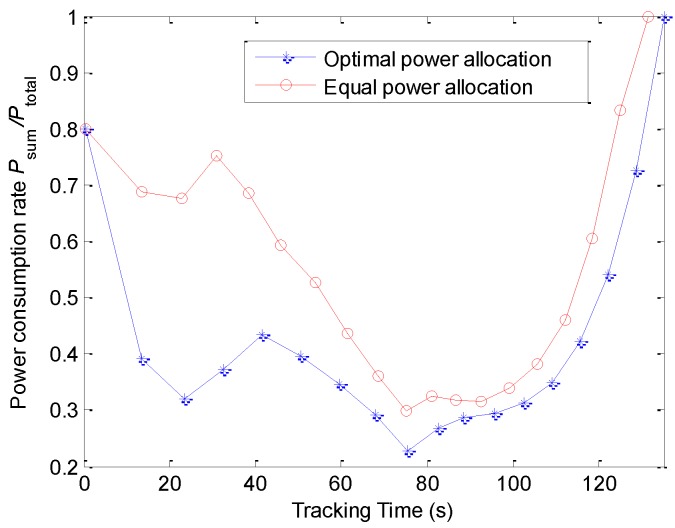
Power resource consumption rate.

The power consumption rate is given in Figure 6. The power consumption rate is defined as the ratio of the total transmitted power *P*_sum_ and the total power *P*_total_ of the radar system. Since the adaptive sampling algorithm is used for the two power allocation methods, it can be seen that they have equal sampling times. Under the condition of the same desired tracking error threshold, the optimal power allocation algorithm can save more power resources than the equal power allocation algorithm, especially between 0 s and 70 s.

*P*_sum_ and *P*_total_ are the total transmitted power and the total power of the radar system, respectively.

**Figure 7:**
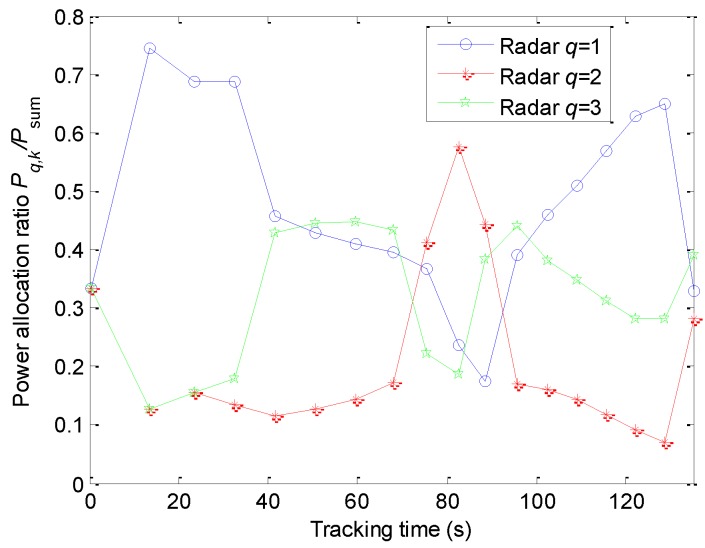
Power allocation ratio.

The power allocation algorithm proposed in this case can also intelligently allocate the power among the radars, and the corresponding simulation results are shown in Figure 7. With the moving of the target, the distance between the target and each radar is time-varying. Attributing to the spatially decentralized radars, the azimuth changing rate and target velocity relative to different radars are different. According to Equations (13) and (14), the measurement values of the range, azimuth, and the Doppler shift can all affect the power allocation, i.e., the power allocation is a comprehensive influence of the three elements. Therefore, the power allocation ratios are changed all the time when the target is moved.

*P_q_*_,*k*_ denotes the transmitted power of the *q*th radar at the *k*th sampling instant.

To examine the tracking performance of the proposed power allocation method, we compare the root MSEs (RMSE) of the state vectors of the two power allocation methods. In this case, the position RMSE is defined by the equation below.

(63) $${\mathrm{RMSE}}_{k}=\sqrt{\frac{1}{N_{\mathrm{MC}}}\sum_{i=1}^{N_{\mathrm{MC}}}\left[{\left(x_{i,k}-{\hat{x}}_{i,k}\right)}^{2}+{\left(y_{i,k}-{\hat{y}}_{i,k}\right)}^{2}\right]}$$

where *N*_MC_ is the number of Monte Carlo trials and it is set to 100 in this paper. $\left({\hat{x}}_{i,k},{\hat{y}}_{i,k}\right)$ is the state estimate of the target at the *i*th trial.

**Figure 8:**
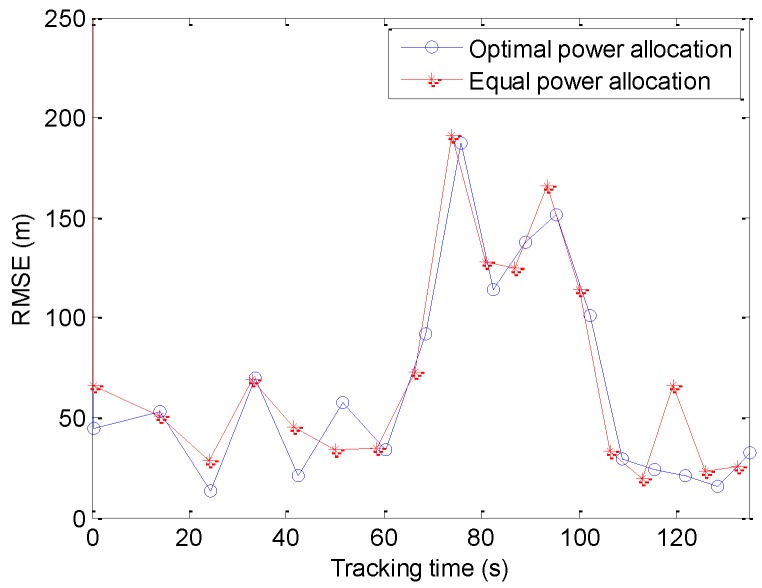
The root mean square error (RMSE) of optimal and equal power allocation.

In Figure 8, although the optimal power allocation consumes less transmitted power, its tracking RMSE is not worse than the equal power allocation. This is because the intelligent increase/decrease of the transmitted power of the radars is certainly better than the simultaneous increase/decrease. In addition, the BCRLB-like no longer provides a lower bound on error performance. Meanwhile, to satisfy the specified confidence level of the CCP, the RCS used in the pre-allocation process is less than the RCS used in the state estimation process. Hence, there is no comparability between the RMSE in Figure 8 and the BCRLB-like in Figure 5a.

RMSE is the root mean square error of the target.

### 5.3. Chance-Constraint Programming

**Figure 9:**
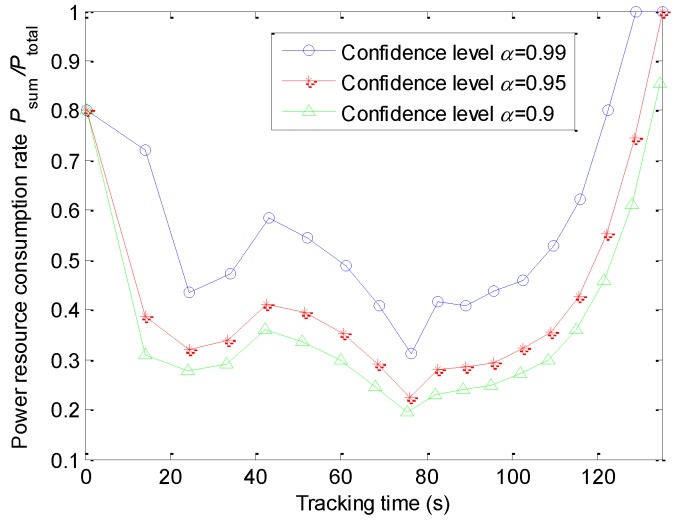
Power consumption of different confidence levels.

To cope with the randomness of RCS, the CCP is utilized in the simulation. Selecting an appropriate confidence level is crucial. Not only the identity, motion state, and number of the targets, but also the target environments and the amount of radar resource are the factors that impact the confidence level. Considering the previously mentioned factors comprehensively, we specify the confidence level *α* as 0.99, 0.95, and 0.9 in Figure 9, respectively. As seen from the simulation results, the power consumption is increased with the rise of the confidence levels.

### 5.4. Target State Estimation with BFG-UKF and IMM-UKF

**Figure 10:**
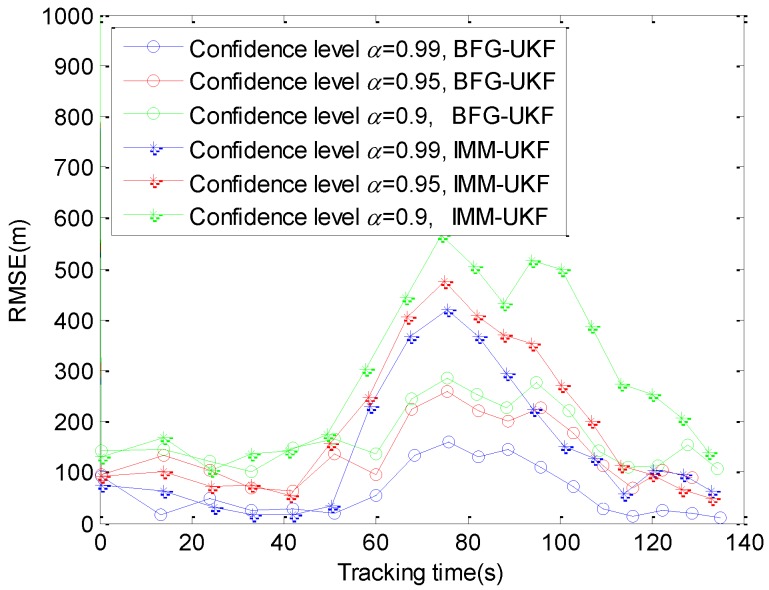
RMSE of different filters.

**Figure 11:**
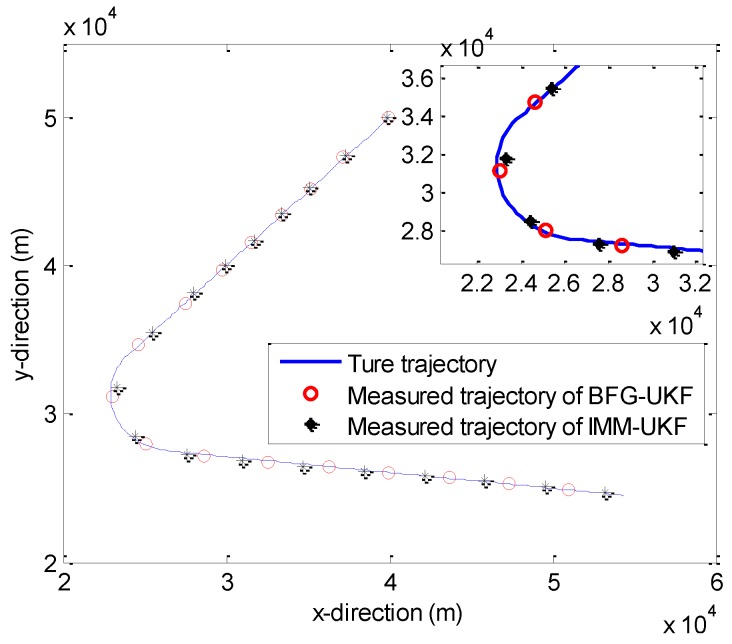
The true and measured trajectories of the maneuvering target.

The regime-dependent motion model is replaced by a single BFG distribution. Consequently, we can use the BFG-UKF to estimate the target state in the previously mentioned three subsections. In this subsection, we compare the RMSE of BFG-UKF and IMM-UKF in Figure 10 and Figure 11.

Where BFG-UKF denotes the UKF combined with BFG.

For BFG-UKF (or IMM-UKF) alone in Figure 10, the RMSEs increase with the decrease of the confidence levels. With the decrease of the confidence levels, the power required to satisfy constraints decreases. Then the tracking performance of BFG-UKF (or IMM-UKF) also gets worse. From 0 s to 60 s and 115 s to 135 s, the tracking effects of BFG-UKF and IMM-UKF are nearly the same. However, between 60 s and 115 s, due to the maneuverability of the target, the estimation performance of the two filters both gets worse. By contrast, the estimation performance of BFG-UKF is better than the estimation performance of IMM-UKF. Now, we give an explanation. Although the traditional UKF is not suitable for tracking the maneuvering target, the BFG is introduced to improve the performance of it and a well simulation is obtained. In addition, in the simulation scenario of this paper, the spatial distribution of radars may result in the inexact calculation of the likelihood function, and then the estimation of the target motion mode is influenced in IMM-UKF. Hence, the simulation results in Figure 10 appear.

Meanwhile, Figure 11 verifies our viewpoint again. In addition, we give a local enlarged drawing to emphasize the simulation results. The confidence level is set as *α* = 0.95 in Figure 11.

The joint resource allocation algorithm of this paper can be extendable to a three-dimensional scenario. In the three-dimensional scenario, the *z*-coordinate is added into target motion equations. In the measurement model, the elevation angle is added. Hence, compared with the two-dimensional scenario, the BCRLB of the elevation angle is added into Equation (16) in the three-dimensional scenario. The predictive BIM of the elevation angle needs to be calculated. Besides the elevation angle, the other predictive BIMs in Equation (40) also need to be derived again. The filter needs to be changed to a three-dimensional form. The joint resource allocation algorithm of the maneuvering target for a three-dimensional scenario is worthy of studying. The formula derivation and simulation are more complicated. For the length of this paper, the content of the three-dimensional scenario is not involved. We will study this part in the future work.

## 6. Conclusions

This paper presents a joint adaptive sampling interval and power allocation scheme based on CCP for MTT in a multiple OAR system. Initially, we develop a general approach to replace the MSD of a maneuvering target with a signal BFG distribution. Based on this, the prior CLRB-like is calculated as a criterion for determining the adaptive sampling interval. Meanwhile, the BCRLB-like, which provides a referenced boundary on the tracking error, is exploited as an aid in performing efficient power allocation for MTT. Afterwards, directing at the randomness of target RCS, we package the deterministic resource allocation model as an uncertain model through CCP conditioned on a specified confidence level. Lastly, the resulting optimization problem is solved through HIOA. Simulation results demonstrate the effect of the adaptive sampling algorithm and the power allocation algorithm, the correctness of HIOA for solving the CCP, and the accuracy of BFG-UKF for estimating the maneuvering target state. As can be seen from the derivation, the resource allocation scheme for a single maneuvering target can be easily generalized to a scenario including multiple maneuvering targets, even the distributed OAR case (consists of *M*_1_ transmitting and *N*_1_ receiving radars). In the future work, we will introduce the BFG approximation into the multiple maneuvering target tracking scenario of the distributed OAR.

## Figures and Tables

**Table 1:** The stochastic simulation.

Step 1. Set *N*′=0;
Step 2. Calculate **Φ***_k_* and **Q**_p,*k*_ according to the BFG approximation;
Step 3. Select a measurement **h***_j_*_,*k*_ (*j*=1, 2, …, *N_H_*) from the measurement set and produce *F*(*T_k_*,**P***_k_*,**h***_j,k_*);
Step 4. If *F*(*T_k_*,**P***_k_*,**h***_j,k_*)≤*η*_1,*k*_, *N*′= *N*′+1;
Step 5. Repeat the third and fourth steps *N_H_* times;
Step 6. Pr{*F*(*T_k_*,**P***_k_*,**h***_k_*)≤*η*_1,*k*_ }= *N*′/*N_H_*.

**Table 2:** The hybrid intelligent optimization algorithm.

(1) Initialize *pop_size* chromosomes, and check the feasibility of the generated chromosomes by the stochastic simulation in Table 1;
(2) Update the chromosomes by crossover and mutation operations in which the feasibility of offspring can be checked by the stochastic simulation in Table 1, and, if they do not satisfy the constraint, correct the chromosomes;
(3) Calculate the objective function values of all the chromosomes;
(4) Compute the fitness of each chromosome according to the objective function values;
(5) Select the chromosomes by spinning the roulette wheel;
(6) Repeat the second to fifth steps for a given number of cycles;
(7) Report the best chromosome as the optimal solution **P***_k_*_,opt_.

## Appendix A

It is assumed that the target motion switches between *N*_m_ models. The evolution of the motion model sequence is modeled by a time-homogenous Markov chain. Then, we define the motion model probabilities.

(A1) $$p_{k}\left(r\right)≜\mathrm{Pr}\left(r_{k}=r\right)$$

where ≜ is a defining operation symbol; *r* = 1, 2, …, *N*_m_. Using the total probability theorem, we get:

(A2) $$p_{k}\left(r\right)=\sum_{j=1}^{N_{m}}p_{jr}p_{k-1}\left(j\right)$$

If the initial model probabilities *p*_1_(*r*) and the transition probabilities *p_ij_* are known, we can determine the *p*_2_(*r*), *p*_3_(*r*), and so on.

Thus, the mean of **ξ***_k_* under model 1 is shown below.

(A3) $$\begin{array}{ll} E[\xi_{k}|\mathrm{model}\;1] & =\sum_{r=1}^{N_{m}}p_{k}\left(r\right)E\left[\xi_{k}|\mathrm{model}\;1,\;r_{k}=r\right]\\ & =\sum_{r=1}^{N_{m}}p_{k}\left(r\right)\left\{F_{k-1}^{r}\left[\xi_{k-1}|\mathrm{model}\;1\right]\right\}\\ & =\left[\sum_{r=1}^{N_{m}}F_{k-1}^{r}p_{k}\left(r\right)\right]E\left[\xi_{k-1}|\mathrm{model}\;1\right] \end{array}$$

Substituting (25) into (A3), we can get the equation below.

(A4) $$E[\xi_{k}|\mathrm{model}\;2]=\Phi_{k-1}E\left[\xi_{k}|\mathrm{model}\;2\right]$$

where

(A5) $$\Phi_{k-1}=\left[\sum_{r=1}^{N_{m}}F_{k-1}^{r}p_{k}\left(r\right)\right]$$

## Appendix B

According to the obtained **Φ**_*k*−1_ in Appendix A, the covariance of **ξ***_k_* under model 2 can be represented by the equation below.

(A6) $$\mathrm{Cov}\left[\xi_{k}|\mathrm{model}\;2\right]=\Phi_{k-1}\mathrm{Cov}\left[\xi_{k-1}|\mathrm{model}\;2\right]{\left(\Phi_{k-1}\right)}^{T}+Q_{p,k-1}$$

It follows from (26) and (A6) that:

(A7) $$Q_{p,k-1}=\mathrm{Cov}\left[\xi_{k}|\mathrm{model}\;1\right]-\Phi_{k-1}\mathrm{Cov}\left[\xi_{k-1}|\mathrm{model}\;1\right]{\left(\Phi_{k-1}\right)}^{T}$$

From (A7), it can be seen that **Φ***_k_*_−1_ and Cov[**ξ***_k_*_−1_|model 1] are both known at time *k*−1. Hence, we need to calculate the first term on the right of the equal sign in (A7). In accordance with the definition of conditional variance, the term Cov[**ξ***_k_*|model 1] can be transformed into the following equation.

(A8) $$\mathrm{Cov}\left[\xi_{k}|\mathrm{model}\;1\right]=E\left[\mathrm{Cov}\left[\xi_{k}|\mathrm{model}\;1,\;r_{k}\right]\right]+\mathrm{Cov}\left[E\left[\xi_{k}|\mathrm{model}\;1,\;r_{k}\right]\right]$$

In the case of model 1, the first term on the right in (A8) can be calculated below.

(A9) $$E[\mathrm{Cov}\left[\xi_{k}|\mathrm{model}\;1,\;r_{k}\right]]=\sum_{r=1}^{N_{m}}p_{k}\left(r\right)\left\{F_{k-1}^{r}\mathrm{Cov}\left[\xi_{k}|\mathrm{model}\;1\right]{\left(F_{k-1}^{r}\right)}^{T}+Q_{k-1}^{r}\right\}$$

Meanwhile, substituting

(A10) $$E[\xi_{k}|\mathrm{model}\;1,\;r_{k}]=F_{k-1}^{r_{k}}E[\xi_{k-1}|\mathrm{model}\;1]$$

into the second term of the right side in (A8), we get

(A11) $$\begin{array}{cc} \mathrm{Cov}\left[E\left[\xi_{k}|\mathrm{model}\;1,\;r_{k}\right]\right] & =\mathrm{Cov}\left[F_{k-1}^{r_{k}}E[\xi_{k-1}|\mathrm{model}\;1]\right]\\ & =E[F_{k-1}^{r_{k}}\left[\xi_{k-1}|\mathrm{model}\;1\right]E{\left[\xi_{k-1}|\mathrm{model}\;1\right]}^{T}{\left(F_{k-1}^{r_{k}}\right)}^{T}]\\ & -E[F_{k-1}^{r_{k}}]E\left[\xi_{k-1}|\mathrm{model}\;1\right]E{\left[\xi_{k-1}|\mathrm{model}\;1\right]}^{T}E{\left[F_{k-1}^{r_{k}}\right]}^{T} \end{array}$$

where

(A12) $$\begin{array}{l} E\left[F_{k-1}^{r_{k}}E\left[\xi_{k-1}|\mathrm{model}\;1\right]E{\left[\xi_{k-1}|\mathrm{model}\;1\right]}^{T}{\left(F_{k-1}^{r_{k}}\right)}^{T}\right]\\ =\sum_{r=1}^{N_{m}}p_{k}\left(r\right)\left\{F_{k-1}^{r}E\left[\xi_{k-1}|\mathrm{model}\;1\right]E{\left[\xi_{k-1}|\mathrm{model}\;1\right]}^{T}{\left(F_{k-1}^{r}\right)}^{T}\right\} \end{array}$$

and

(A13) $$E[F_{k-1}^{r_{k}}]=\Phi_{k-1}$$
